# Geographic Variation of Chronic Kidney Disease Prevalence: Correlation with the Incidence of Renal Cell Carcinoma or Urothelial Carcinoma?

**DOI:** 10.1155/2015/427084

**Published:** 2015-10-28

**Authors:** Yit-Sheung Yap, Kai-Wen Chuang, Chun-Ju Chiang, Hung-Yi Chuang, Sheng-Nan Lu

**Affiliations:** ^1^Division of Nephrology, Department of Internal Medicine, Yuan's General Hospital, Kaohsiung 802, Taiwan; ^2^Division of Hepatogastroenterology, Department of Internal Medicine, Kaohsiung Chang Gung Memorial Hospital and Chang Gung University, Kaohsiung 833, Taiwan; ^3^Graduate Institute of Epidemiology and Preventive Medicine, College of Public Health, National Taiwan University, Taipei 100, Taiwan; ^4^Taiwan Cancer Registry, Taipei 100, Taiwan; ^5^Department of Public Health, Kaohsiung Medical University, Kaohsiung 807, Taiwan; ^6^Department of Environmental and Occupational Medicine, Kaohsiung Medical University Hospital, Kaohsiung 807, Taiwan

## Abstract

*Background*. The aim of this study is to evaluate whether geographic variations in the prevalence of late-stage chronic kidney disease (CKD) exist and are associated with incidence rates of renal cell carcinoma (RCC), upper tract urothelial carcinoma (UTUC), or lower tract urothelial carcinoma (LTUC). *Methods*. Prevalence rates of late-stage CKD for 366 townships (*n* > 30) in Taiwan were calculated for 1,518,241 and 1,645,151 subjects aged 40 years or older in years 2010 and 2009, respectively. Late-stage CKD prevalence in year 2010 was used as a training set and its age-adjusted standardized morbidity rates (ASMR) were divided into three groups as defined <1.76%, 1.76% ≤ ASMR < 2.64%, and ≥2.64%, respectively. Year 2009, defined as the validation set, was used to validate the results. *Results*. The ASMR of late-stage CKD in years 2010 and 2009 were 1.76%, and 2.09%, respectively. Geographic variations were observed, with notably higher rates of disease in areas of the central, southwestern mountainside, and southeastern seaboard. There were no significant differences among different combined risk groups of RCC, UTUC, and LTUC incidence. *Conclusion*. The substantial geographic variations in the prevalence of late-stage CKD exist, but are not correlated with RCC, UTUC, or LTUC incidence.

## 1. Introduction

Chronic kidney disease (CKD) has emerged as a worldwide public health problem and burden [1]. According to the 2014 United States Renal Data System (USRDS) report, Taiwan had the greatest prevalence and the third greatest incidence of end stage renal disease (ESRD) [2]. The ESRD population, 0.3% of the total population, consumes over 6% of the total annual budget for the National Health Insurance (NHI) Program on dialysis [3]. Among many reasons contributing to the high prevalence and incidence of ESRD, the high prevalence of CKD may be one of the most important factors [4].

While there has been amply documented evidence on the geographic variation of ESRD in many countries, studies of regional patterns of CKD prevalence at the township level in a country are lacking [2]. In Taiwan, previous reports have documented that high incidence of ESRD or CKD mainly occur in central and southern regions [5, 6]. A report even documented that there was also geographic variation of late-stage CKD at the township level in a county [7]; nevertheless, these reports only investigated the geographic variations of CKD at the county level or focused on a district.

Patients with CKD may have increased risk of overall cancer [8]. Besides, there is consistent evidence of an excess risk of renal cell carcinoma (RCC), upper tract urothelial carcinoma (UTUC), and lower tract urothelial carcinoma (LTUC) among patients with ESRD [8, 9]. Patients with ESRD may have a higher risk for RCC or urothelial carcinoma (UC) because of sharing common risk factors, such as smoking, using Chinese herbal concoctions containing aristolochic acid, and abusing analgesics [10, 11]. In fact, the incidences of CKD as well as RCC or UC are high in Taiwan and are mainly distributed in the southern region [5, 12, 13]. Nevertheless, to date, few studies have investigated these cancer risks in relation to less severe forms of CKD by using a geographical distribution method.

We hypothesized that there would be substantial differences in the late-stage CKD prevalence across geographic areas and the late-stage CKD prevalence would be associated with township-specific RCC or UC incidence rates. Hence, the present study aimed, firstly, to determine the distribution of late-stage CKD prevalence using geographical information and grade the risk for different regional patterns and, secondly, to evaluate whether geographic variation in the prevalence of late-stage CKD would be correlated with RCC, UTUC, and LTUC incidence rates.

## 2. Materials and Methods

### 2.1. Study Areas and Study Population

The study areas were all 368 townships in Taiwan, composed of 5 municipalities, 3 cities, and 14 counties. This study comprised residents who participated in the APSP and were aged 40 years or older in the years 2010 and 2009. The Adult Preventive Service Program (APSP) is a free physical examination program, which has been organized by the Administration of Health Promotion since 1997, and provides laboratory test and general physical examination annually for adult persons aged over 65 years and poliomyelitis patients aged over 35 years, and once every three years for persons aged between 40 and 64 years. In the years 2010 and 2009, a total of 1,544,828 and 1,669,436 subjects eligible for the health examination were enrolled. The database was linked to the Department of Household Registration, Ministry of the Interior, for obtaining the residing township of each participant. We excluded subjects aged below 40 years and without the information of resident townships (*n* = 26528/24207 in years 2010/2009), and two study townships because of less than or equal to 30 examinees located at small outlying islands (Dongyin (Lienchiang) and Wuqiu (Kinmen), *n* = 29/28 and 30/50 in years 2010/2009). Hence, the finalized study populations were 1,518,241 and 1,645,151 individuals, respectively, in 366 townships, in the years 2010 and 2009. The study protocol was approved by the Institutional Review Board of Chang Gung Memorial Hospital, Taiwan. Since the individual identification and personal information were removed by the Health Promotion Authority before analysis, the Institutional Review Board approved the waiver of the requirement of written consent from all participants.

### 2.2. Measurements and Definitions

The sole available laboratory value analyzed was creatinine, and the estimated glomerular filtration rate (eGFR) was calculated. The eGFR was estimated using the four variable Modification of Diet in Renal Disease (MDRD) Study formula [14] as follows: 186 × (serum creatinine (mg/dL))^−1.154^  × (age)^−0.203^  × (0.742 if female). CKD definitions were based on Kidney Disease Quality Outcomes Initiative (KDOQI) guidelines [15]. We classified patients with stage 3b to stage 5 as late-stage CKD. Stage 3b was defined as eGFR of 30–44 mL/min/1.73 m^2^, stage 4 as eGFR of 15–29 mL/min/1.73 m^2^, and stage 5 as eGFR of <15 mL/min/1.73 m^2^.

### 2.3. Mapping the Risk of Geographic Distribution of Late-Stage CKD Prevalence

Township-specific prevalence rates of late-stage CKD in year 2010 were used as the training set. We calculated the age-adjusted standardized morbidity rates (ASMR) for each township and ASMR was expressed as a percentage. All townships were then divided into three groups based on ASMR value of <1.76%, 1.76% ≤ ASMR < 2.64%, and ≥2.64%, respectively, for each significant and nonsignificant category. For obtaining the consistency of the results, year 2009 was used as a validation set. ASMR of each township was calculated, and all townships were also divided into three groups in each significant and nonsignificant category based on cut-off point of years 2010. After validation, ASMR groups of both years were combined and classified into two broad risk categories, determined as indefinite risk with significant ASMR groups in one of the years and definite risk with nonsignificant or significant ASMR groups for both years. For significant ASMR in only one year, risk groups were defined as significantly low (ASMR < 1.76%), high (1.76% ≤ ASMR < 2.64%), or higher (ASMR ≥ 2.64%) in either year. For significant ASMR groups of both years, risk groups were defined as low, high, higher, and highest, respectively. The risk classifications were as follows: low = ASMR < 1.76% for both years; high = ASMR < 1.76% in either year and 1.76% ≤ ASMR < 2.64% in another year/1.76% ≤ ASMR < 2.64% for both years; higher = 1.76% ≤ ASMR < 2.64% in either year and ASMR ≥ 2.64% in another year; highest = ASMR ≥ 2.64% for both years; indeterminate = ASMR < 1.76% in either year and ASMR ≥ 2.64% in another year.

### 2.4. Renal Cell Carcinoma and Urothelial Carcinoma Incidence Rates

RCC, UTUC, and LTUC incidence rates for both years were provided by the Central Cancer Registry using the International Classification of Diseases, Ninth Revision (ICD-9), and the revision codes for the following: RCC (189); UTUC (189.1–189.2); and LTUC (188, 189.3). Incidence rates were calculated as the number of incident cases divided by the total Taiwanese population in each township of both years and expressed as a rate per 100,000 population.

### 2.5. Statistical Analysis

ASMR of late-stage CKD was presented as mean and standard deviation. Paired* t-*test was used for comparing the region-level or county-level ASMR between years 2010 and 2009 at 95% confidence interval (CI). To calculate ASMR, Taiwan populations in both years were used as standard population. ASMR of 1.76% (first cut point) was defined as ASMR of all townships by using population of year 2010; and ASMR of 2.64% (second cut point) represented the 1.5 times of 1.76%. We performed goodness-of-fit analysis to compare the late-stage CKD prevalence in townships with the national prevalence of year 2010 for both years. Additionally, to examine the association between late-stage CKD and RCC and UC, comparison of different combined risk groups of RCC or UC incidences were analyzed with the Kruskal-Wallis test. Using Quantum geographic information systems software (QGIS) version 1.8.0, township level of late-stage CKD was plotted according to categorization of ASMR. A value of *P* < 0.05 was considered statistically significant. The goodness-of-fit analysis was carried out using SAS software (Version 9.3; SAS Institute Inc., Cary, NC); for assessment of the others, SPSS software (Version 19; SPSS Inc., Chicago, IL) was used.

## 3. Results

### 3.1. ASMR of Late-Stage Chronic Kidney Disease

The mean prevalence and ASMR of late-stage CKD in years 2010 and 2009 were 6.4% and 6.9%, and 1.76% and 2.09% respectively. In Table 1, for the ASMR of late-stage CKD in regional patterns, the eastern region and outlying islands had the highest ASMR among these 5 regions in year 2010 and 2009 respectively, established as 2.31% and 2.22%. Based on the classification in county level, the ASMR of late-stage CKD ranged from 1.50% in Kinmen county and New Taipei city to 2.70% Taitung county in year 2010; and ranged from 1.69% in Chiayi county to 2.84% in Lienchiang county for year 2009. Due to large sample sizes, there were also significant mean differences for year comparison across many counties. Keelung city and Taipei city in northern region had a difference of more than 0.5%; and the largest significant difference was 0.58% in Taipei city.

### 3.2. Maps of Late-Stage CKD Prevalence according to ASMR and Goodness-of-Fit Test

**Figure 1:**
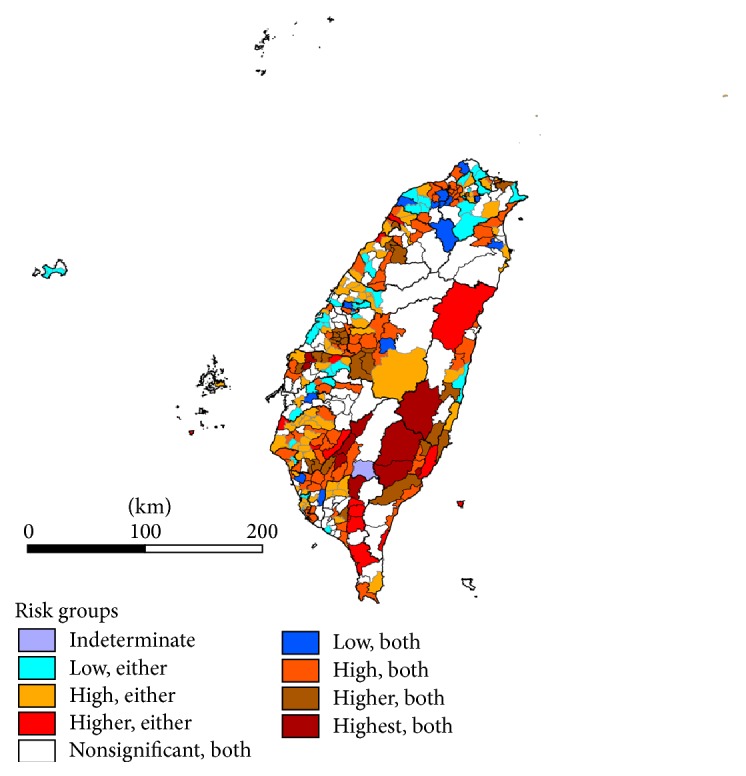
Risk categorization after combining age-adjusted standardized morbidity rates (ASMR, expressed as percentage) groups of both year 2010 and year 2009.  Indeterminate = chronic kidney disease prevalence of townships being zero (unable to perform goodness-of-fit test). Significant ASMR in either year: Low, either = Significant ASMR < 1.76% in either year; High, either = Significant 1.76% ≤ ASMR < 2.64% in either year; Higher, either = Significant ASMR ≥ 2.64% in either year. Nonsignificant of ASMR in both years: Nonsignificant, both = ASMR is not significant comparing with whole study populations in both years. Significant ASMR in both years: Low, both = ASMR < 1.76% for both years; High, both = ASMR < 1.76% in either year and 1.76% ≤ ASMR < 2.64% in another year/1.76% ≤ ASMR < 2.64% for both years; Higher, both = 1.76% ≤ ASMR < 2.64% in either year and ASMR ≥ 2.64% in another year; Highest, both = ASMR ≥ 2.64% for both years.

After combination of significant ASMR groups of both years, the consistence of ASMR groups was identified as 57.6% (ASMR < 1.76%: 15/132 = 11.4%; 1.76% ≤ ASMR < 2.64%: 52/132 = 39.4%; ASMR ≥ 2.64%: 9/132 = 6.8%) (Table not shown).  Figure 1 display the geographic variation in late-stage CKD prevalence after validation and combination according to varied significant or non-significant ASMR groups of both years. For significant ASMR in only one year, townships with high (1.76% ≤ ASMR < 2.64%) or higher (ASMR ≥ 2.64%) risk were primarily scattered at northwestern, central and southwestern areas, noted as counties of Hsinchu, Miaoli, Taichung, Yunlin, Tainan, Kaohsiung and Pingtung. For significant ASMR in both years, 15 townships had significantly lower rates of prevalence, with ASMR < 1.76%. In contrast, 82 townships were of significantly high risk. Higher as well as the highest risk group were noted in 26 and 9 townships respectively, mainly distributed at central (Changhua, Nantou and Yunlin); and southwestern mountainside areas (Tainan, Kaohsiung and Pingtung). Parts of townships were distributed at the southeastern seaboard, noted as Taitung. Nine townships with highest risk were as follow: Lunbei (Yunlin), Baojhong (Yunlin), Jiasian (Kaohsiung), Shanlin (Kaohsiung), Namasia (Kaohsiung), Sandimen (Pingtung), Jhuosi (Hualien), Haiduan (Taitung), Yanping (Taitung).

### 3.3. Correlation of ASMR of Late-Stage CKD with RCC, UTUC and LTUC Incidence Rates

In Table 2, the median incidence rates of RCC in different combined risk groups ranged from 2.97 to 5.14 (expressed as a rate of per 100,000 population). For UTUC and LTUC, the median incidence rates ranged from 4.22 to 5.73 and 7.30 to 11.08 respectively. Unexpectedly, there were no significant differences among different combined risk groups of RCC, UTUC and LTUC incidence.

## 4. Discussions

Using data from a large sample of adult subjects who were enrolled in a prevalence study from across all townships in Taiwan, we demonstrated substantial geographic variation in the prevalence of late-stage CKD. However, there was no significant correlation present between the prevalence of late-stage CKD with RCC or UC incidence rates.

Prior studies have established that the prevalence of CKD varies widely from country to country because of the discrepancies in age, ethnic groups, known risk factors to CKD, survey policies, and equations of eGFR calculation [4, 16, 17]. Similarly, the variation of CKD prevalence has also existed in Taiwan in different times. In the present study, the prevalence and ASMR of late-stage CKD showed as high as 7% and 2% respectively, higher than the CKD prevalence of previous studies [17, 18]. There are several possible explanations for these differences. First, the majority of the recruited subjects were limited to those aged 40 years or older, instead of 20 years or older. Second, the increasing numbers of late-stage CKD patients could be attributed to an aging society due to improvement of public health and medical care. Besides, the CKD prevalence in the elderly age group increased markedly when compared to a younger age group [5]. Third, the previous CKD prevalence was believed to be widely underestimated because of limited sampling methods and sample size, and low awareness in these populations, especially with subjects of low socioeconomic and educational status [4, 11].

Geographic variations of ESRD incidence have been widely studied in different nations [2, 19, 20]. Lower socioeconomic level, lack of access to medical care, and incidence of underlying kidney diseases were among the possible causes [21–23]. A previous study has even illustrated that lower population physician density instead of chronic morbidities was more significantly related to regional variations of ESRD incidence [23]. In Taiwan, geographic differences of ESRD or CKD were also reported, and the high-risk areas were located at central and southern regions [5–7]. However, these reports only investigated the geographic variations of CKD in county level or focused on a district. To our knowledge, countywide rates may mask important patterns by averaging high and low areas, known as ecological bias. For instance, a high-risk area around a mountainside in a rural region would be lost in a county-level map, as shown in our study. Additionally, it is unknown whether regional variations in CKD prevalence explains regional variation in ESRD incidence. This speculation could probably be validated in a report from the Taiwan Renal Registry, which demonstrated the area with high ESRD incidence as over 400 per million population (pmp) in a county level overlapping with the high and higher risk regions of CKD in our study [6]. Besides, an investigation in Japan also confirmed that regional differences in CKD prevalence may underlie the variation in ESRD incidence rates [22]. Taken together, the identification of geographic heterogeneity in CKD prevalence has important implications. It is because CKD is not only associated with incline of ESRD incidence, but also related to an increased risk of CVD and mortality, and early identification of subjects at risk has the potential to reduce the burden of these complications [4, 24].

Previous studies established that patients with CKD might have increased risk of kidney and urothelial cancer [8, 9]. CKD is a state of chronic inflammation, leading to transformation of normal cells to tumor cells, tumor proliferation, and angiogenesis. It is postulated that the uremic toxins in patients with CKD may further accentuate the mitogenesis and cell differentiation in malignant tumors [25]. Of note, UC could be attributed to potential exposure of urinary carcinogens, such as the use of aristolochic acid in Chinese herbal medicine, which is also a cause of chronic interstitial nephritis and progression to end stage kidney disease [26]. To our knowledge, herbal drugs are very popular in southern Taiwan, particularly in rural areas. Nevertheless, contrary to expectations, in the present study, no significant correlations were found between late-stage CKD prevalence and RCC or UC incidence rates. One potential explanation for this finding is that RCC or UC is a rare disease in the general population, and we have limited statistical power for analyses by only using a two-year study interval of cancer incidence rates. Moreover, it is postulated that RCC is more prevalent among dialysis patients, particularly those with long-term dialysis history, but not for late-stage CKD subjects [27]. The association between dialysis and RCC risk is likely due to complications of chronic kidney disease such as development of acquired cystic kidney disease [9].

The primary strength of our study is the inclusion of subjects from all regions of the country giving a better representation of late-stage CKD prevalence than studies from one geographic region or city [2, 5–7]. Additionally, we used laboratory test results for analysis instead of using diagnostic code; this produced more precise and accurate information. However, our study has certain limitations. First, we measured GFR only once and thus may have misclassified persons with only acute kidney injury. Nevertheless, this misclassification would bias the observed results toward the null. Second, the majority of the subjects were restricted to adult person aged 40 years or older; the prevalence of CKD in younger individuals has not been estimated. However, prevalence of late-stage CKD is very low for people younger than 40 years [5]; hence, the results of this study did not affect the policy for CKD prevention. Third, laboratory data were measured by different hospital/clinical/laboratory centers and different laboratory equipment; thus measurement bias such as random bias and systematic bias might exist; however, these biases did not influence the categorization of risk groups because the consistency of grading was high, and no townships with significant ASMR had marked discrepancy of grading.

In conclusion, this is the first study of the epidemiological status of township-specific prevalence of late-stage CKD. The present study demonstrated the presence of substantial geographic variation in the prevalence of late-stage CKD. However, we found no correlation between late-stage CKD prevalence with RCC or UC incidence rates. Further investigations are warranted to evaluate the other specific reasons behind the geographical variations. If the factors contributing to late-stage CKD can be identified, improving prevention and treatment services in these high prevalence areas should be a priority.

## Figures and Tables

**Table 1:** Comparison of ASMR of late-stage chronic kidney disease between years 2010 and 2009, by county or city groups.

County/city (township numbers)	Total subject numbers		ASMR^a^		Mean difference% (95% CI)	*P* value
	Year 2010	Year 2009	Year 2010	Year 2009		
Northern region	607462	660776	1.73 (0.35)	2.06 (0.39)	−0.34 (−0.42~−0.26)	<0.001

Keelung City (7)	23063	28420	1.69 (0.20)	2.23 (0.22)	−0.53 (−0.69~−0.38)	<0.001
Taipei City (12)	128014	142552	1.81 (0.18)	2.39 (0.26)	−0.58 (−0.80~−0.35)	<0.001
New Taipei City (29)	198986	210902	1.50 (0.30)	2.00 (0.34)	−0.50 (−0.63~−0.37)	<0.001
Taoyuan County (13)	115789	123098	1.58 (0.23)	1.87 (0.35)	−0.29 (−0.41~−0.17)	<0.001
Hsinchu City (3)	21114	24901	2.15 (0.14)	2.63 (0.20)	−0.48 (−0.84~−0.12)	0.028
Hsinchu County (13)	29834	34271	1.95 (0.36)	2.04 (0.40)	−0.09 (−0.41~0.23)	0.552
Miaoli County (18)	41476	50386	1.85 (0.40)	1.92 (0.44)	−0.07 (−0.24~0.09)	0.361

Central region	378563	411753	1.83 (0.36)	2.15 (0.64)	−0.32 (−0.43~−0.21)	<0.001

Taichung City (29)	155445	171526	1.75 (0.18)	1.98 (0.25)	−0.23 (−0.33~−0.13)	<0.001
Changhua County (26)	109803	117762	1.75 (0.27)	2.15 (0.68)	−0.40 (−0.64~−0.17)	0.002
Nantou County (13)	45661	48965	1.99 (0.31)	2.32 (0.43)	−0.33 (−0.56~−0.11)	0.008
Yunlin County (20)	67654	73500	1.95 (0.58)	2.30 (1.00)	−0.35 (−0.71~0.01)	0.055

Southern region	470025	504705	1.88 (0.48)	2.20 (0.79)	−0.32 (−0.45~−0.18)	<0.001

Chiayi City (2)	22496	23921	1.70 (0.08)	1.85 (0.10)	−0.15 (−0.33~0.04)	0.063
Chiayi County (18)	58134	60455	1.57 (0.26)	1.69 (0.43)	−0.13 (−0.31~0.05)	0.155
Tainan City (37)	151786	154324	1.80 (0.31)	2.27 (0.30)	−0.47 (−0.58~−0.37)	<0.001
Kaohsiung City (38)	174684	200524	2.02 (0.53)	2.36 (0.71)	−0.34 (−0.61~−0.07)	0.015
Pingtung County (33)	62965	65481	2.01 (0.58)	2.33 (1.22)	−0.22 (−0.64~0.19)	0.278

Eastern region	46539	52370	2.31 (0.83)	2.19 (0.66)	0.12 (−0.18~0.41)	0.423

Yilan County (12)	49186	46246	1.82 (0.37)	2.11 (0.34)	−0.29 (−0.58~0.01)	0.055
Hualien County (13)	26361	30661	1.82 (0.64)	2.16 (0.70)	−0.34 (−0.69~0.01)	0.057
Taitung County (16)	20178	21709	2.70 (0.77)	2.21 (0.66)	0.49 (0.11~0.87)	0.016

Outlying islands	15652	15547	1.87 (0.74)	2.22 (1.16)	−0.36 (−1.14~0.43)	0.341

Penghu County (6)	8039	8527	1.92 (0.48)	2.29 (0.81)	−0.36 (−1.18~0.46)	0.307
Kinmen County (5)	7398	6766	1.50 (0.03)	1.78 (0.26)	−0.29 (−0.60~0.02)	0.062
Lienchiang County (3)	215	254	2.37 (1.50)	2.84 (2.42)	−0.47 (−8.42~7.52)	0.823

ASMR = age-adjusted standardized morbidity rates; CI = confidence interval.

^a^Data presented as mean (standard deviation) in percentage.

**Table 2:** Kruskal-Wallis test for different combined risk groups of renal cell carcinoma or urothelial carcinoma incidence.

Level of risk	Risk groups	*N*	RCC	UTUC	LTUC
Indefinite	Low	40	3.59 (0–10.54)	4.70 (0–16.38)	7.30 (0–28.07)
	High	77	3.99 (0–14.95)	4.22 (0–32.77)	8.30 (0–36.04)
	Higher	15	5.14 (0–17.51)	5.73 (0–14.28)	11.08 (3.90–40.84)

Definite	Nonsignificant	101	3.66 (0–14.45)	4.37 (0–27.01)	9.00 (0–27.28)
	Low	15	4.12 (0–10.15)	4.54 (0–17.32)	8.72 (0–30.74)
	High	82	3.67 (0–20.03)	5.35 (0–35.96)	8.83 (0–59.93)
	Higher	26	2.97 (0–13.47)	4.98 (0–14.06)	8.63 (0–13.28)
	Highest	9	4.93 (0–7.64)	5.66 (0–12.17)	7.40 (1.85–12.57)

*P* value			0.291	0.484	0.410

*N* = numbers of townships; RCC = renal cell carcinoma; UTUC = upper tract urothelial carcinoma; LTUC = lower tract urothelial carcinoma.

Data are presented as median (range) and express a rate per 100,000 population.

## Abbreviations

CKD: Chronic kidney disease
RCC: Renal cell carcinoma
UTUC: Upper tract urothelial carcinoma
LTUC: Lower tract urothelial carcinoma
ASMR: Age-adjusted standardized morbidity rates
USRDS: United States Renal Data System
ESRD: End stage renal disease
NHI: National Health Insurance
UC: Urothelial carcinoma
APSP: Adult Preventive Service Program
eGFR: Estimated glomerular filtration rate
MDRD: Modification of Diet in Renal Disease
KDOQI: Kidney Disease Quality Outcomes Initiative
QGIS: Quantum geographic information systems.
